# Type B lymphomatoid papulosis

**DOI:** 10.11604/pamj.2018.30.138.15295

**Published:** 2018-06-18

**Authors:** Ahmed Bouhamidi, Badreddine Hassam

**Affiliations:** 1 Department of Dermatology, Ibn Sina University Hospital, Rabat, Morocco

**Keywords:** Lymphomatoid papulosis, cutaneous T-Cell Lymphoma, mycosis fungoides

## Image in medicine

A 28-year-old woman with no notable medical history. Who presented with a 3-month history of pruritic erythematous purplish papules at the axilla, elbow folds, flanks, inner thigh and popliteal. On physical examination, there were multiple papular lesions with irregular pigmentation at the axilla (A), associated with maculopapular erythematous plaques on elbow folds, flanks, inner thigh and popliteal (B). There was no lymphadenopathy or mucosal involvement. Skin biopsy was in favor of type B lymphomatoid papulosis with the presence of rare large cells, with CD30 expression confined to the cell membrane and Golgi region (C, D). All the staging investigations were normal. The diagnosis of type B lymphomatoid papulosis was retained and the patient treated with topical corticosteroids with a good improvement, she is under regular monitoring. Lymphomatoid papulosis is characterized by erythematous papulonodular lesions, often evolving into a crust and spontaneously disappear in a few weeks, leaving a depressed and sometimes pigmented scar. Type B lymphomatoid papulosis histology consists of an infiltrate of lymphocytes with cerebriform nuclei, the infiltrate includes large cells that can express the CD30 antigen. lymphomatoid papulosis has a good prognosis but can be associated with or progress to malignancy. Therefore, it is important to monitor these patients.

**Figure 1:**
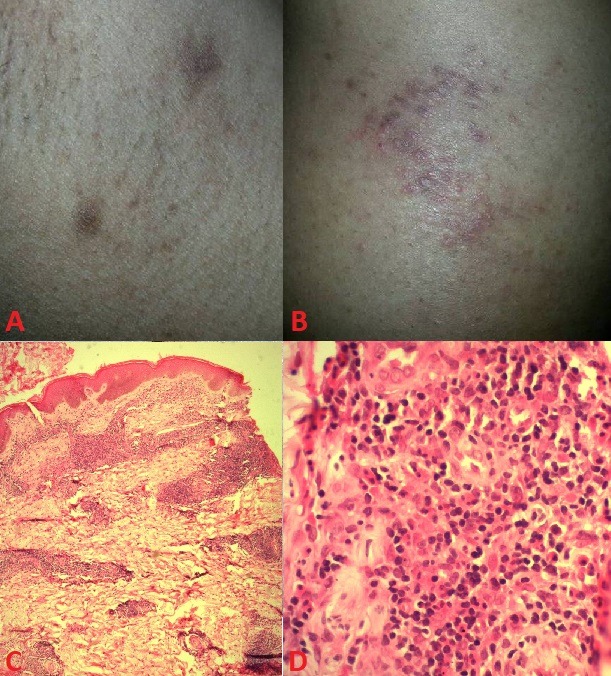
A) papular lesions with irregular pigmentation on the axilla; B) erythematous papules on the flanks; C) large lymphocytes, with CD30 expression confined to the cell membrane and Golgi region (H&E, original magnification x100); D) atypical large lymphocytes, with CD30 expression confined to the cell membrane and Golgi region (H&E, original magnification x400)

